# Long-term follow-up of survival after passive containment surgery in dilated cardiomyopathy

**DOI:** 10.1016/j.amsu.2022.104241

**Published:** 2022-07-31

**Authors:** Fredrik Bredin

**Affiliations:** Section of Cardiothoracic Surgery, Department of Molecular Medicine and Surgery, Karolinska Institutet, Stockholm, Sweden

The failing heart undergoes numerous structural and functional changes often referred to as ventricular remodeling [1,2]. The Acorn Cor Cap™ cardiac support device (CSD) is a mesh-like polyester fabric with bidirectional compliance. The CSD was developed to be positioned around the failing heart, thereby facilitating reversed remodeling of the heart, which includes reshaping of the heart from a dilated spherical shape to an ellipsoidal shape [3].

We previously reported our early and mid-term results on the application of CSD in patients with ischemic or idiopathic (i.e., non-ischemic) cardiomyopathy (CMP) [4,5]. Mann et al. previously reported the five-year results of the Acorn Trial that demonstrated that application of the CSD may be beneficial for patients suffering from heart failure symptoms despite treatment with optimal pharmacological therapy [6]. No longtime studies for this group of patients have earlier been published. This study group is therefore unique and long -time follow up is complete and therefore the results are of interest although the cohort is small and there is no control group. Due to difficulties in identifying selection criteria the current use of the CSD have almost vanished.

In this paper, we present the long-term survival outcomes of patients with CMP that underwent CSD implantation at our institution between 2001 and 2006.

Between 2001 and 2006, 20 patients with ischemic (n = 10) or idiopathic (n = 10) CMP received the CSD either as the sole procedure (n = 3) or in conjunction with other open-heart surgery procedures (n = 17). The study was approved by the local ethical committee at the Karolinska University Hospital (approval file number 01–159) and written consent was obtained from all patients. Inclusion criteria has been described earlier [5].

Patients preoperative characteristics, additional surgery and post operative survival time are presented in Table 1. Patient files were used to determine cause of death. The Kaplan-Meier method was used to calculate cumulative survival. All patients survived the surgical procedure. Follow-up was complete and one patient (Patient 15) was alive at the completion of the study.

**Table 1:** Patients preoperative characteristics, additional surgery and postoperative survival time.

Patient	Age years	Gender F/M	Etiology Isch/Idio	NYHA class	LVEF %	LVEDmm	Additional surgery	Survival time months
1	66	M	Isch	3	15	67	CABGx1	25
2	69	M	Isch	3	25	60	CABGx2	192
3	50	F	Idio	2	30	70	–	168
4	58	M	Idio	4	15	73	MVR	204
5	44	M	Idio	3	30	73	MVR	80
6	61	M	Isch	2	30	67	CABGx2	198
7	78	M	Idio	3	10	62	–	76
8	72	M	Isch	3	15	80	CABGx3	71
9	72	M	Isch	3	20	60	CABGx3	49
10	42	M	Idio	4	20	81	–	2
11	32	M	Idio	2	40	67	MVR	202
12	56	F	Idio	3	15	80	MVR	20
13	51	M	Isch	2	30	68	CABGx3	142
14	58	M	Idio	2	25	77	MVR	50
15	66	M	Idio	3	15	77	MVR	Alive
16	59	M	Idio	3	25	82	MVR	60
17	72	M	Isch	3	20	69	CABGx3	2
18	70	M	Isch	3	20	85	CABGx2	66
19	63	M	Isch	3	25	72	CABGx3	60
20	65	M	Isch	3	20	69	CABGx3	72

CABG: coronary artery bypass grafting; F: female; Idio: idiopathic; Isch: ischemic; LVED: left ventricular end diastolic diameter; LVEF: left ventricular ejection fraction; M: male; MVR: mitral valve repair; NYHA: New York Heart Association.

Patient 11 underwent a heart transplantation 11 years after CSD application; 5 years after transplantation, the patient died because of a suspected coronavirus disease-19 infection. Terminal heart failure was the sole or a major contributing cause of death in all other patients. Postoperative survival time and etiology of CMP are presented in Fig. 1.

**Fig. 1:**
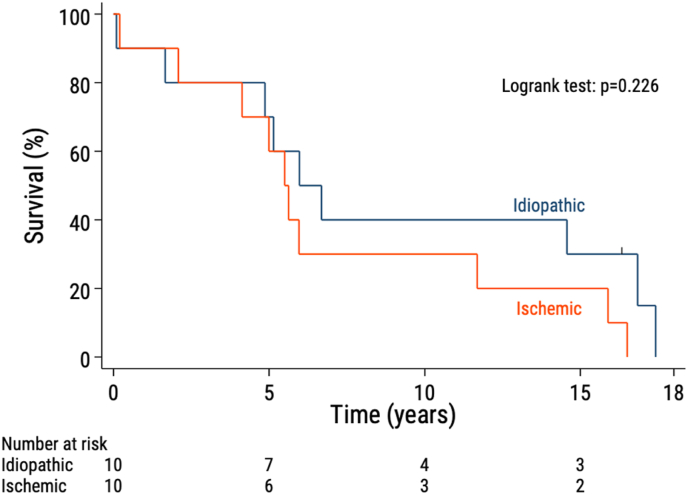
The observed survival in patients with idiopathic (blue panel) respectively ischemic (red panel) CMP after application of the CSD. (For interpretation of the references to colour in this figure legend, the reader is referred to the Web version of this article.)

The term reversed remodeling has been used to describe the mechanics leading to improvements in clinical manifestations and prognosis in heart failure patients [7].

The findings in the present study indicate that for patients with dilated CMP, receiving the CSD as the sole procedure or in conjunction with other heart surgery procedures show unpredictable effects regarding facilitating reversed remodeling. Patient prognosis was not affected by the etiology of CMP (ischemic vs idiopathic). These results differ from our findings on the short-term follow-up of CMP patients but is in accordance with our findings on mid-term follow-up in these patients [4,5]. The definitive conclusion of this study is that for patients with dilated CMP irrespective of etiology application of the CSD shows no clear beneficial effects.

## Ethical approval

This study was approved by the local ethics committee at the Karolinska University Hospital (approval file number 01–159).

## Sources of funding for your research

This study was supported by an independent donation from Mr F Lundberg and funds from the Mats Kleeberg Foundation.

## Author contribution

Fredrik Bredin is the sole author contributing to this paper.

## Registration of research studies

1. Name of the registry:
2. Unique Identifying number or registration ID:
3. Hyperlink to your specific registration (must be publicly accessible and will be checked):

## Guarantor

The Guarantor is the one or more people who accept full responsibility for the work and/or the conduct of the study, had access to the data, and controlled the decision to publish.

## Consent

Written consent was obtained from all patients.

## Declaration of competing interest

I declare that I have no known competing financial interests or personal relationships that could have appeared to influence the work reported in this paper.
